# 5-Azacytidine and Resveratrol Enhance Chondrogenic Differentiation of Metabolic Syndrome-Derived Mesenchymal Stem Cells by Modulating Autophagy

**DOI:** 10.1155/2019/1523140

**Published:** 2019-05-12

**Authors:** K. Marycz, J. M. Irwin Houston, C. Weiss, M. Röcken, K. Kornicka

**Affiliations:** ^1^Department of Experimental Biology, Wroclaw University of Environmental and Life Sciences, 50-375 Wroclaw, Poland; ^2^International Institute of Translational Medicine, Jesionowa 11, 55-114 Wisznia Mała, Poland; ^3^PferdePraxis Dr. Med. Vet. Daniel Weiss, Postmatte 14, CH-8807 Freienbach, Switzerland; ^4^Faculty of Veterinary Medicine, Equine Clinic-Equine Surgery, Justus-Liebig-University, Giessen 35392, Germany

## Abstract

Recently, metabolic syndrome (MS) has gained attention in human and animal metabolic medicine. Insulin resistance, inflammation, hyperleptinemia, and hyperinsulinemia are critical to its definition. MS is a complex cluster of metabolic risk factors that together exert a wide range of effects on multiple organs, tissues, and cells in the body. Adipose stem cells (ASCs) are multipotent stem cell population residing within the adipose tissue that is inflamed during MS. Studies have indicated that these cells lose their stemness and multipotency during MS, which strongly reduces their therapeutic potential. They suffer from oxidative stress, apoptosis, and mitochondrial deterioration. Thus, the aim of this study was to rejuvenate these cells *in vitro* in order to improve their chondrogenic differentiation effectiveness. Pharmacotherapy of ASCs was based on resveratrol and 5-azacytidine pretreatment. We evaluated whether those substances are able to reverse aged phenotype of metabolic syndrome-derived ASCs and improve their chondrogenic differentiation at its early stage using immunofluorescence, transmission and scanning electron microscopy, real-time PCR, and flow cytometry. Obtained results indicated that 5-azacytidine and resveratrol modulated mitochondrial dynamics, autophagy, and ER stress, leading to the enhancement of chondrogenesis in metabolically impaired ASCs. Therefore, pretreatment of these cells with 5-azacytidine and resveratrol may become a necessary intervention before clinical application of these cells in order to strengthen their multipotency and therapeutic potential.

## 1. Introduction

Metabolic syndrome in humans (MetS) and horses (EMS) is more and more frequently diagnosed endocrine disorder all over the world, especially in well-developed countries [1, 2]. It occurs as a result of diet based on carbohydrate overload along with limited physical activity and genetic predisposition [1–3] and is characterized by fasting hyperleptinemia and hyperinsulinemia. Although obesity in MetS is recognized as a diagnostic factor, recent data suggests that severe obesity is not required for EMS diagnosis [4]. Finally, MetS and EMS culminate in vascular dysfunction, which in the course of MetS leads to the development of cardiovascular diseases and in EMS to *laminitis*. Moreover, epidemiological and experimental evidence supports the link between obesity, hyperleptinemia, hyperinsulinemia, and osteoarthritis (OA) development [5]. It is hypothesized that hyperinsulinemia combined with increased levels of serum insulin-like growth factors (IGFs) and abundant adipokine secretion from adipose tissue may trigger OA development and joint degradation. Moreover, during MetS and EMS, systemic and local inflammation is associated with infiltration of adipose tissue with inflammatory cells, such as macrophages, lymphocytes, and mast cells [6, 7].

As it was recently demonstrated, cartilage dysfunction in metabolic syndrome may be caused by systemic hyperlipidemia, hypercholesterolemia, or hyperglycaemia [8, 9]. It was also reported that lipid diffusion into the joints through systemic circulation and synovial fluid is linked to cartilage matrix protein oxidation and increased synovial permeability [10, 11]. Moreover, high glucose and cholesterol levels were shown to be strongly associated with catabolic and anabolic metabolism of chondrocytes and synovium as well as activation and ectopic bone formation in the early stage collagenase-induced OA [12, 13].

Recently, MetS and EMS have been shown to be associated with cytophysiological deterioration of adipose-derived stem cells (ASCs) [14, 15]. As we have previously shown, in the course of EMS, ASCs are characterized by impaired multipotency and immunomodulatory effect, which severely limits their proregenerative properties [16–18]. Due to their multipotency, nowadays, ASCs are widely applied in regenerative medicine, including OA treatment [19]. Collection of adipose tissue is easier and less invasive when compared to bone marrow biopsies, and isolated cells can be rapidly expanded *in vitro* which make them an attractive tool in cell-based therapies [20]. What is more, they exert a wide range of immunomodulatory effects due to the inhibition of CD4+ T cells, CD8+ T cells, B cells, and natural killer (NK) cells and activation of regulatory T cells (Treg) [21]. Additionally, ASCs promote macrophages polarization into immunosuppressive M2 type, which supports their application in the treatment of proinflammatory diseases, including metabolic syndrome [22]. We have also shown that ASCs are effective in the treatment of musculoskeletal disorders in small and large animals [23, 24]. Proregenerative properties of ASCs are partially explained by secretion of extracellular microvesicles (ExMVs) which improve intercellular signaling and support tissue regeneration [25, 26]. ExMVs contain a broad spectrum of cytokines, adipokines, hormones, and soluble growth factors that play a pivotal role in tissue regeneration [27]. Recently, ASC-derived ExMVs have been shown to contain high levels of proteins related to chondrogenic differentiation, including vascular endothelial growth factor B (VEGFB), hypoxia-inducible factor-1*α* (HIF-1*α*), fibroblast growth factor-2 (FGF-2), transforming growth factor-*β*1, and bone morphogenetic protein 2 (BMP-2) [28]. However, our recent data showed that metabolic syndrome severely impairs the chondrogenic differentiation potential of ASCs obtained from EMS horses through increased DNA methylation, excessive oxidative stress, and mitochondrial dysfunction [16, 17]. Therefore, we postulate that *ex vivo* pretreatment of ASC derived from EMS horses (ASC_EMS_) with 5-azacytidine (AZA) and resveratrol (RES) may become distinct form of cellular pharmacotherapy able to reverse phenotype and improve multipotency of deteriorated cells. Our previous study revealed that *ex vivo* application of AZA reversed the cytophysiological impairment of aged ASCs by epigenetic modifications and reduction of oxidative stress [29]. AZA treatment increased the mRNA levels of ten-eleven translocation methylcytosine dioxygenases (TET) and the B-cell lymphoma 2 (BCL-2)/bcl-2-like protein 4 (BAX) ratio, resulting in improved ASCs' viability. On the other hand, RES, a natural polyphenol, has been shown to play a critical role in the regulation of cell fate and longevity *via* the activation of 5′ AMP-activated protein kinase (AMPK), forkhead box O3 (FOXO-3), and sirtuin-1 (SIRT1) genes [30]. In addition to its antioxidant activity, RES has been shown to reduce the inflammatory response and increase mitochondrial biogenesis by upregulating eNOS, which is associated with the SIRT1 pathway [31, 32].

In this study, we evaluated the chondrogenic differentiation potential of ASC_EMS_ treated *ex vivo* with the combination of AZA and RES. We examined the expression of genes and levels of proteins involved in the formation of extracellular matrix, oxidative stress, autophagy, mitochondrial biogenesis, and dynamics.

## 2. Materials and Methods

All reagents used in this experiment were purchased from Sigma-Aldrich (Poland), unless indicated otherwise.

### 2.1. Classification of Animals

Horses were age-matched (mixed sex, 9–14 years; mean ± SD, 11.2 ± 1.7 years) and assigned into two groups: healthy (ctrl) horses (*n* = 5; 2 female, 3 male) and EMS (*n* = 5; 2 female, 3 male). The detailed characterization of animals which participated in the experiments is shown in Table 1. Animals were assigned to proper group based on the following parameters: (i) body weight, (ii) body condition score (BCS) and cresty neck scoring system (CNS), (iii) visual and X-ray examination of the hoof capsule, (iv) resting insulin and leptin levels, and (v) combined glucose-insulin test (CGIT) as described previously [33].

### 2.2. ASC Isolation

Adipose tissue was harvested with the approval of the Local Ethical Committee in Wroclaw (84/2018). Approximately, 2 g of adipose tissue was harvested from the horses' tail base. Next, tissue samples were placed in sterile Hank's balanced salt solution (HBSS). Cells were isolated under aseptic conditions following the previously described protocol [34]. Briefly, specimens were cut into small pieces, minced, and digested in collagenase type I solution (1 mg/mL) for 40 min at 37°C. Following digestion, samples were centrifuged (1200 × g, 10 min.). The remaining cell pellet was resuspended in a culture medium and transferred to a culture flask. In order to perform the experiments, cells were passaged three times using TrypLE™ Express (Life Technologies).

### 2.3. Evaluation of Cell Phenotype

The expression of CD44, CD45, and CD90 surface antigens in isolated cells was investigated with flow cytometry. In order to perform immunophenotyping, ASCs were harvested using TrypLE Express solution (Life Technologies), washed with HBSS, and resuspended to a total concentration of 5 × 10^5^ cell/mL. Cell suspension was incubated with specific monoclonal antibodies preconjugated with fluorescein isothiocyanate (FITC, Abcam) (anti-CD44, R&D Systems, MAB5449; anti-CD45, Novus Biologicals, NB1006590APC; anti-CD90, Abcam, ab225). Data was acquired for at least ten thousands of stained cells by Becton Dickinson FACSCalibur flow cytometer and analyzed using CellQuest Pro or Flowing Software.

### 2.4. Multipotency Assay

To determine a potential for osteogenic, adipogenic, and chondrogenic differentiation of ASC, isolated cells were cultured in STEMPRO Osteogenesis Differentiation Kit and STEMPRO Adipogenesis Differentiation Kit (Life Technologies) according to the manufacturer's protocol. To perform the test, the cells were seeded in a 24-well plate at a density of 1 × 10^4^ cells in 500 *μ*L of medium per well. The media were changed every two days. ASC differentiation into adipocytes lasted 11 days, while osteogenesis and chondrogenesis lasted 10 days. Osteocytes were stained with 1% solution of Alizarin Red to visualize the osteogenic-specific extracellular mineralized matrix. Intracellular lipid droplets formed during adipogenesis were stained with Oil Red O while proteoglycan-rich matrix in chondrocytes was visualized using Safranin O staining.

### 2.5. Cell Culture

Culture medium consisted of DMEM with 4,500 mg/L glucose supplemented with 10% fetal bovine serum (FBS) and 1% of penicillin-streptomycin (PS). Culture medium was changed every three days. Cells were passaged after reaching 90% confluence. After third passage, ASCs were seeded onto 24-well plates at a density of 2 × 10^4^ per well. When cells attached, regular culture medium has been exchanged for medium supplemented with 0.5 *μ*M of AZA and 0.05 *μ*M of RES. Pretreatment lasted for 24 hours, then the experimental medium was replaced by STEMPRO Osteogenesis Differentiation Kit (Life Technologies). Cells were cultured in osteogenic medium for five then and after were subjected to analysis. ASCs isolated from healthy controls have been labelled as CTRL, while from EMS individuals EMS. ASCs from EMS horses pretreated with AZA/RES have been entitled as the EMS EXP group.

### 2.6. BrdU Assay

Cell proliferation rate was assessed using commercially available BrdU Cell Proliferation ELISA Kit (Abcam). The method is based on BrdU incorporation into newly synthesized DNA strands of proliferating cells. All procedures were performed in accordance with the manufacturer's protocol. In order to perform the test, the cells were seeded onto microtiter plates (96-well plate) at an initial concentration of 1 × 10^4^ cells in 100 *μ*L of chondrogenic media per well. For the assay, 20 *μ*L of diluted BrdU reagent was added directly to the medium, then incubated for 24 h, at 37°C in a CO_2_ incubator condition. Thereafter, cells were fixed and permeabilized, and the DNA was denaturated by provided fixing solution. Incorporated reagent was detected using anti-BrdU monoclonal detector primary antibody. Subsequently, cells were incubated with secondary antibodies (horseradish peroxidase-conjugated goat anti-mouse IgG). The color reaction was performed by using peroxidase chromogenic substrate TMB (tetramethylbenzidine). Absorbance was measured at 450 nm using a spectrophotometric microtiter plate reader (BMG Labtech).

### 2.7. Scanning (SEM) and Transmission Electron (TEM) Microscopy

SEM was used to determine detailed cells morphology in accordance with the protocol described previously [17]. Cells were fixed with 4% paraformaldehyde (PFA) and dehydrated with ethanol (concentrations from 50 to 100%, every 5 min) as described elsewhere [35, 36]. Samples were sprinkled with gold (ScanCoat 6, Oxford) and images were taken using SE1 detector, at 10 kV of filament tension.

In order to visualize ultrastructure of cells, TEM analysis was performed as described previously [17]. Briefly, cells were fixed with 2.5% glutaraldehyde and incubated with 1% osmium tetroxide/HBSS for 2 hours. Dehydration was performed in acetone-graded series. Samples were embedded in Agar Low Viscosity Resin Kit (Agar Scientific Ltd., Essex, UK). To collected ultrathin sections (80 nm), cooper grids were used. Uranyl acetate and lead citrate were used for the attainment of a contrast. Auriga 60 Zeiss STEM was at 20 kV filament tension.

### 2.8. Immunofluorescence

Visualization of mitochondria was performed using the MitoRed staining. Cells were incubated with MitoRed (dilution 1 : 1000) for 30 minutes at 37°C and fixed with PFA. Nuclei were counterstained with DAPI diluted 1 : 1000 in HBSS for 5 minutes.

In order to visualize intracellular protein localization, investigated cells were treated as described previously [17]. Membranes were permeabilized with 0.5% X-100 (20 min, room temperature) and incubated with blocking buffer (10% goat serum, 0.2% Tween-20 in HBSS). Staining was performed with the following primary antibodies: anti-LAMP2 (1 : 500, Abcam), PINK1 (1 : 200, Biorbyt, orb331233), PARKIN (1 : 200, Novus, NB100-91921) diluted in HBSS containing 1% goat serum, and 0.2% Tween-20. Atto-488-conjugated secondary antibodies (dilution 1 : 1000, Abcam) were applied for 1 hour in a dark. Cells' nuclei were counterstained with DAPI dye for 5 minutes.

F-Actin filaments were visualized using staining with Phalloidin–Atto 565 in accordance with the manufacturer's protocol. The observations were performed, and images were taken using confocal microscope microscope (Observer Z1 Confocal Spinning Disc V.2 Zeiss with live imaging chamber). Pictures were analyzed using the ImageJ software.

Analysis of mitochondrial net morphology was performed in MiNA software [37].

### 2.9. Oxidative Stress Factors

Reactive oxygen species (ROS) were determined by incubating cells with an H2DCF-DA (Life Technologies). Superoxide dismutase (SOD) activity was evaluated using a commercially available SOD Assay kit (Sigma-Aldrich). Nitric acid (NO) concentration was measured using the Griess Reagent Kit (Life Technology). All processes were performed according to the manufacturer's instructions.

### 2.10. Flow Cytometry Analysis

All flow cytometry analyses were performed after 24 hours of chondrogenic differentiation as described previously [38]. Cells were incubated with anti-5-mC antibody (Abcam, ab73938) and anti-histone H3 (Abcam, ab8898) with 10% goat serum. Then, cells were costained with Alexa 488 goat anti-mouse secondary antibodies (1 : 500, Alexa Fluor 488, Abcam).

Flow cytometry analysis was performed to assess a mitochondrial membrane potential. Cells were collected and incubated with JC1 (1 mM) (Life Technologies) for 30 min at 37°C. The cells were washed and analyzed by FACSCalibur flow cytometer. Data was acquired for at least 5 thousands of stained cells and analyzed using CellQuest Pro software.

### 2.11. Isolation of Proteins and Western Blotting

In order to determine the presence of specific proteins in investigated cells by western blotting, protein isolation was performed. Cells were detached from culture dishes and homogenized in RIPA buffer containing protease inhibitor cocktail. The samples were centrifuged at 14,000 × g for 20 min at 4°C. Supernatants were collected and transferred into new tubes. The protein concentration was determined by the Pierce™ BCA Protein Assay Kit (Life Technologies, Warsaw, Poland). Cell lysates (30 *μ*g of proteins for each sample) were separated on SDS-PAGE gels at 100 V for 90 min in Tris/glycine/SDS buffer and transferred onto PVDF membranes (Bio-Rad) using a transfer apparatus at 100 V for 1 h at 4°C in Tris/glycine buffer. After transfer, the membranes were washed with Tris/NaCl/Tween buffer (TBST). After washing, the overnight blocking with 5% nonfat milk in TBST at 4°C was performed. Afterwards, the membranes were washed with TBST and incubated with primary antibody against *β*-actin (Sigma-Aldrich, A5441), mitofusin-1 (MFN-1) (Biorbyt, orb11040,), and mitochondrial fission factor (MFF) (Biorbyt orb325479) at a dilution of 1 : 500 for 2 hours followed by incubation with ALP-conjugated secondary antibodies. After 2 h incubation with secondary antibodies, the membranes were washed with TBST and detected using the BCIP®/NBT-Purple Liquid Substrate for 15 min. The reaction was stopped by washing the membranes with water.

### 2.12. Quantitative Real-Time Reverse Transcription Polymerase Chain Reaction (RT-PCR).

Analysis of gene expression was performed after the fifth day of differentiation as described elsewhere [39]. Total RNA was extracted using the phenol-chloroform method as originally described by Chomczynski and Sacchi [40]. RNA quality and quantity were determined with spectrophotometry (Epoch, Biotek). Genomic DNA was digested performed using DNase I, RNAase-free (Life Technologies) while complementary DNA (cDNA) synthesis was performed using a RevertAid RT Reverse Transcription Kit (Life Technologies). Primer concentration equaled 500 nM, and their sequences are shown in Table 2. Reactions were carried out using the SensiFAST SYBR & Fluorescein Kit (Bioline) and a CFX Connect™ Real-Time PCR Detection System (Bio-Rad). Relative gene expression analysis (Qn) was evaluated in relation to the GAPDH as a housekeeping gene using the ΔΔCt method. Moreover, the ratio of Bcl-2/BAX expression was determined by dividing ΔΔCt values of those genes.

### 2.13. Coculture of ASC with RAW 264.7

Coculture of ASC with RAW 264.7 was performed as described previously [38]. RAW 264.7 was at a density of 1 × 10^6^ cells/mL and seeded onto a 24-well plate. Next, lipopolysaccharide (LPS, 1 *μ*g/mL) was added to the culture media for another 24 h. At the same time, ASCs after the fifth day of chondrogenic differentiation (4 × 10^4^) were added to culture wells. After 24 hours of coculture, media were collected and the cells were lysed by adding TRI Reagent.

### 2.14. Statistical Analysis

Statistical analysis was performed as described previously [38] using the unpaired Student *t*-test with the GraphPad Prism 5 Software (La Jolla, USA). Differences with a probability of *p* < 0.05 were considered significant. Statistical significance indicated as asterisk (^∗^) when comparing the result to ASC_EMS_ and as number sign (#) when comparing to ASC_CTRL_.

## 3. Results

### 3.1. Immunophenotyping and Multipotency Assay

**Figure 1:**
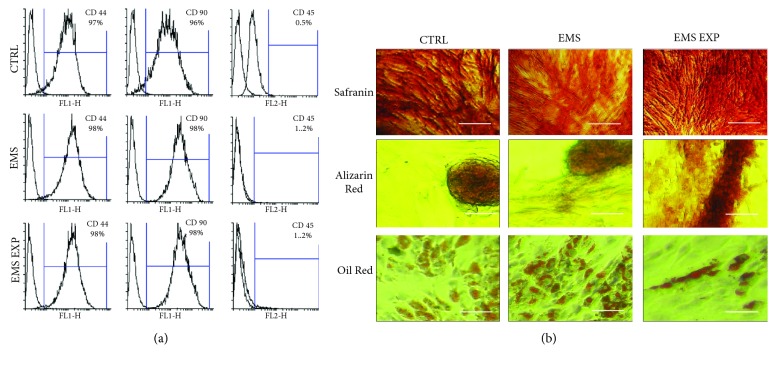
Immunophenotyping and multipotency assay. Expression of CD44, CD90, and CD 45 was investigated with flow cytometry (a). Effectiveness of differentiation was established by specific staining (b). Safranin stained proteoglycans formed during chondrogenic differentiation while Alizarin Red stained extracellular mineralized matrix formed in the course of osteogenic differentiation. Intracellular lipid droplets formed during adipogenesis were stained with Oil Red dye. Scale bar: 500 *μ*M.

Both ASC isolated from healthy and EMS-diagnosed horses exhibited typical phenotypic characteristics of adipose-tissue mesenchymal stem cells: expression of surface proteins such as CD44 and CD90 and the lack of CD45 (Figure 1(a)). Furthermore, AZA/RES treatment did not affect the cells' surface antigen profile. Moreover, multilineage differentiation potential was confirmed by positive results of adipogenesis, chondrogenesis, and osteogenesis as visualized by specific staining (Figure 1(b)).

### 3.2. Effectiveness of Chondrogenic Differentiation

**Figure 2:**
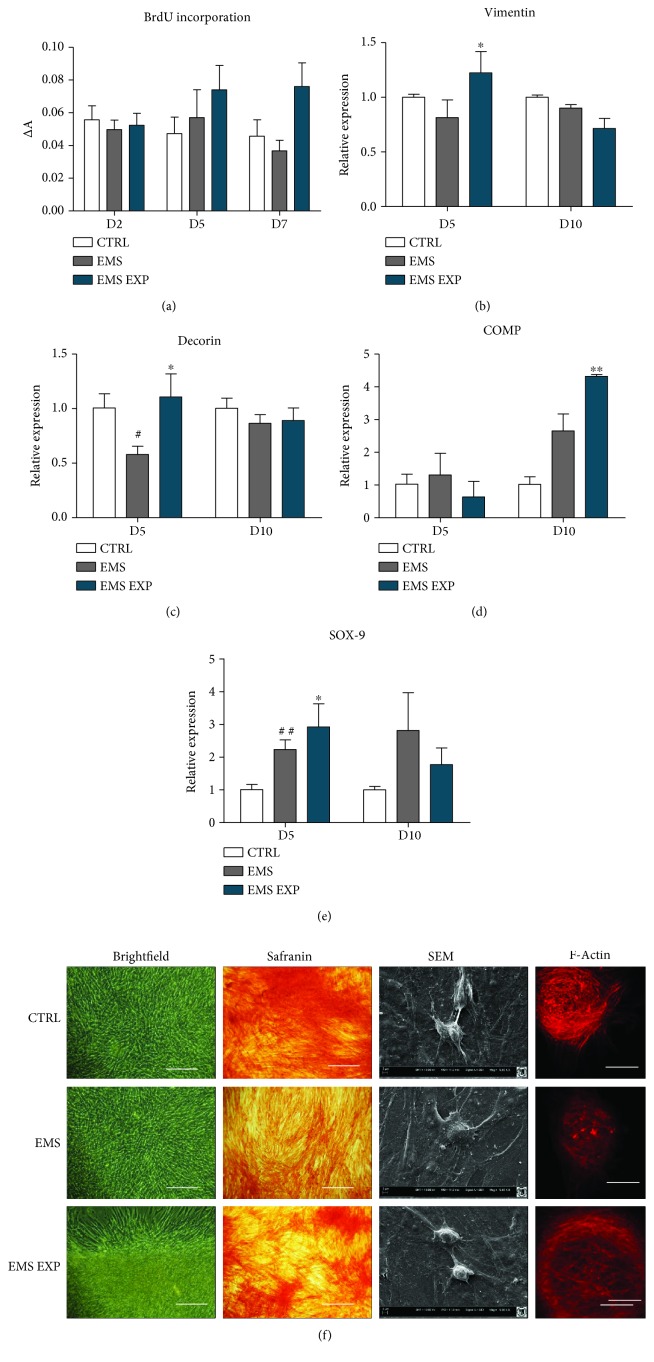
Effectiveness of chondrogenic differentiation. Prior to experiments, cells were pretreated with AZA/RES for 24 hours, then the experimental medium was replaced by osteogenic differentiation medium. Cells were cultured in that medium for five days and after were subjected to further analysis. In order to investigate proliferative activity of cells, BrdU assay was performed (a). Expression of chondrogenesis-related genes, including vimentin (b), decorin (c), COMP (d), and SOX-9 (e) was analyzed by RT-PCR. Cells in culture were visualized using light microscope, safranin staining, SEM, and confocal microscope (F-actin) (f). Scale bars: brightfield: 250 *μ*M, confocal 500 *μ*M. Results expressed as mean ± S.D. Statistical significance indicated as an asterisk (^∗^) when comparing the result to ASC_EMS_ and as a number sign (#) when comparing to ASC_CTRL_. ^#^ and ^∗^*p* < 0.05; ^##^ and ^∗∗^*p* < 0.01.

The proliferation rate of equine ASC (EqASC) was evaluated within 7 days of chondrogenic differentiation using the BrdU proliferation assay. Magnitude of an absorbance is proportional to a quantity of BrdU incorporation during DNA synthesis in proliferating cells, which is a direct indication of the proliferation. We observed no significant differences in the proliferation of both ASC_CTRL_ and ASC_EMS_. Interestingly, proliferation of ASC_EMS_ treated with AZA/RES significantly increased at day seven in comparison to ASC_EMS_ (Figure 2(a)). In order to evaluate chondrogenesis effectiveness, the expression of vimentin (Figure 2(b)), decorin (Figure 2(c)), COMP (Figure 2(d)), and SOX-9 (Figure 2(e)) was established on the 5th and 10th days of differentiation. Vimentin, decorin, and SOX-9 expression was increased after AZA/RES treatment at day 5, while COMP at day 10. Brightfield photographs including Safranin staining confirmed the formation of proteoglycans (Figure 2(f)). Moreover, using the SEM formation of chondrogenic nodules, it was visualized. Obtained photographs indicated that AZA/RES treatment resulted in the formation of greater nodules. F-Actin staining confirmed SEM data as enlarged nodules was noted in the AZA/RES group.

### 3.3. Evaluation of Apoptosis in EqASC

**Figure 3:**
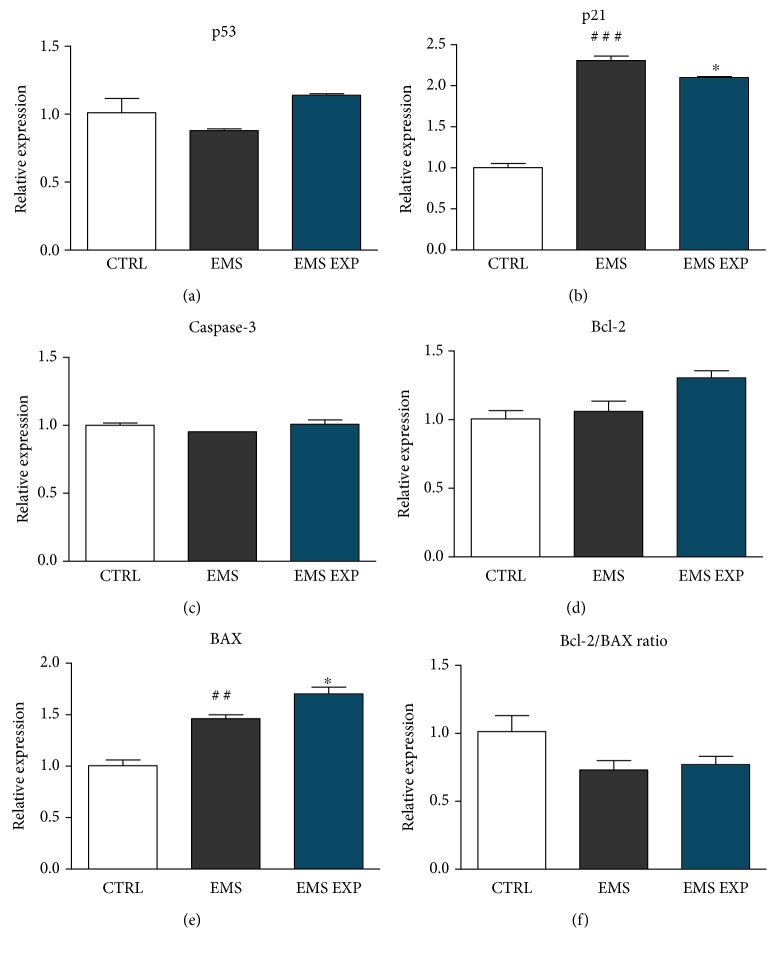
Evaluation of apoptosis. Prior to experiments, cells were pretreated with AZA/RES for 24 hours, then the experimental medium was replaced by osteogenic differentiation medium. Cells were cultured in that medium for five days and after were subjected to further analysis. In order to evaluate apoptosis in cells, expression of p53 (a), p21 (b), caspase-3 (c), Bcl-2 (d), and BAX (e) was analyzed by RT-PCR. Furthermore, Bcl-2/BAX ratio was calculated using relative expression values (f). Results expressed as mean ± S.D. Statistical significance indicated as an asterisk (^∗^) when comparing the result to ASC_EMS_ and as a number sign (#) when comparing to ASC_CTRL_. ^∗^*p* < 0.05; ^##^*p* < 0.01; ^###^*p* < 0.001.

Apoptosis was assessed by analysis of p53, caspase-3, Bcl-2, and BAX expression in ASC isolated from healthy and EMS-diagnosed horses, as well as in ASC_EMS_ after AZA/RES treatment. Quantitative analysis of those genes revealed no statistical significant differences of p53 (Figure 3(a)), caspase-3 (Figure 3(c)), and Bcl-2 (Figure 3(d)) expression between investigated groups. AZA/RES treatment resulted in decreased expression of p21 (Figure 3(b)). Interestingly, there was significantly higher amount of BAX mRNA level in ASC_EMS_ treated with AZA/RES in comparison both the EMS and CTRL group (*p* < 0.05) (Figure 3(e)). Additionally, there was no significant differences of BCl-2/BAX ratio in the experimental groups (Figure 3(f)).

### 3.4. Investigation of Oxidative Stress Factors

**Figure 4:**
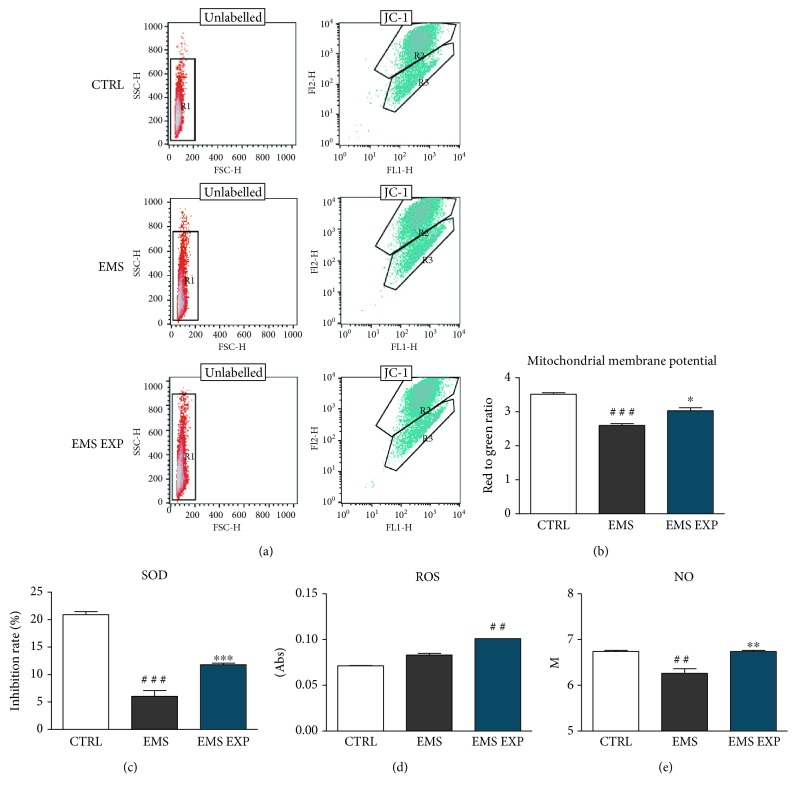
Investigation of oxidative stress factors. Prior to experiments, cells were pretreated with AZA/RES for 24 hours, then the experimental medium was replaced by osteogenic differentiation medium. Cells were cultured in that medium for five days and after were subjected to further analysis. MMP in cells was established by flow cytometry using JC-1 assay (a, b). AZA/RES treatment improved MMP in ASC_EMS_. Furthermore, extracellular levels of SOD (c), ROS (d), and NO (e) were investigated using commercially available assays based on spectrophotometric measurements. Increased values of SOD, ROS, and NO were noted in the experimental group. Results expressed as mean ± S.D. Statistical significance indicated as an asterisk (^∗^) when comparing the result to ASC_EMS_ and as a number sign (#) when comparing to ASC_CTRL_. ^∗^*p* < 0.05; ^##^ and ^∗∗^*p* < 0.01; ^###^ and ^∗∗∗^*p* < 0.001.

To estimate oxidative stress, concentrations of ROS and NO, an activity of SOD, as well as mitochondrial membrane potential (MMP) were evaluated in ASC_CTRL_, ASC_EMS_, and ASC _EMS EXP_. Representative graphs from JC-1 analysis are shown in Figure 4(a). MMP was lower in the EMS group in comparison with the ASC_CTRL_ group, while it significantly increased in ASC_EMS EXP_ (*p* < 0.05) (Figure 4(b)). SOD activity was reduced in EMS, while the enzyme activity significantly increased in the EMS EXP group (*p* < 0.001) (Figure 4(c)). Both ROS (Figure 4(d)) and NO (Figure 4(e)) concentrations were higher in ASC_EMS EXP_ than in the ASC_EMS_ group. Moreover, the level of NO in ASC_EMS EXP_ was comparable to the control group.

### 3.5. Assessment of Endoplasmic Reticulum (ER) Stress during Differentiation

**Figure 5:**
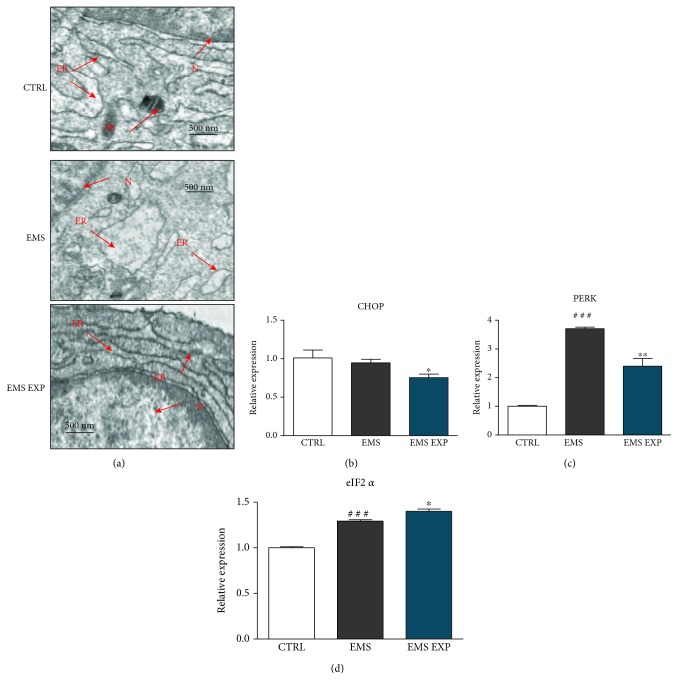
Assessment of ER stress during differentiation. Prior to experiments, cells were pretreated with AZA/RES for 24 hours, then the experimental medium was replaced by osteogenic differentiation medium. Cells were cultured in that medium for five days and after were subjected to further analysis. ER structure in cells was visualized by TEM (a). ASC_EMS_ were characterized by swallowed ER lumen, while in the experimental group, ER was properly developed. Expression of ER stress-related genes CHOP (b) and PERK (c) was diminished in cells pretreated with AZA/RES. Interestingly, expression of eIF2*α* (d) was enhanced in ASC_EMS_ and EMS_EXP_. Abbreviations: ER: endoplasmic reticulum, N: nucleus, Mt: mitochondrion. Results expressed as mean ± S.D. Statistical significance indicated as an asterisk (^∗^) when comparing the result to ASC_EMS_ and as a number sign (#) when comparing to ASC_CTRL_. ^∗^*p* < 0.05; ^###^*p* < 0.001.

ER stress during chondrogenesis was evaluated using the TEM microscopy (Figure 5(a)). In ASC_CTRL_, ER presented typical, well developed, and rough morphology. Contrarily, ER in ASC_EMS_ displayed abnormal morphology including fragmentation, swollen lumen, and disintegration. ASC_EMS_ after treatment with AZA/RES exhibited higher integration of ER with the presence of many ribosomes in comparison to the ASC_EMS_, without the presence of vacuoles. Additionally, RT-PCR confirmed increased ER stress in the ASC_EMS_ group during differentiation. Significant downregulation of CHOP (Figure 5(b)) (*p* < 0.05) and PERK (Figure 5(c)) (*p* < 0.01) was observed in ASC_EMS_ treated with AZA/RES, while eIF2*α* (Figure 5(d)) expression was strongly increased (*p* < 0.05). Obtained results suggest that AZA/RES treatment attenuates ER stress.

### 3.6. Investigation of Autophagy during Early Chondrogenesis

**Figure 6:**
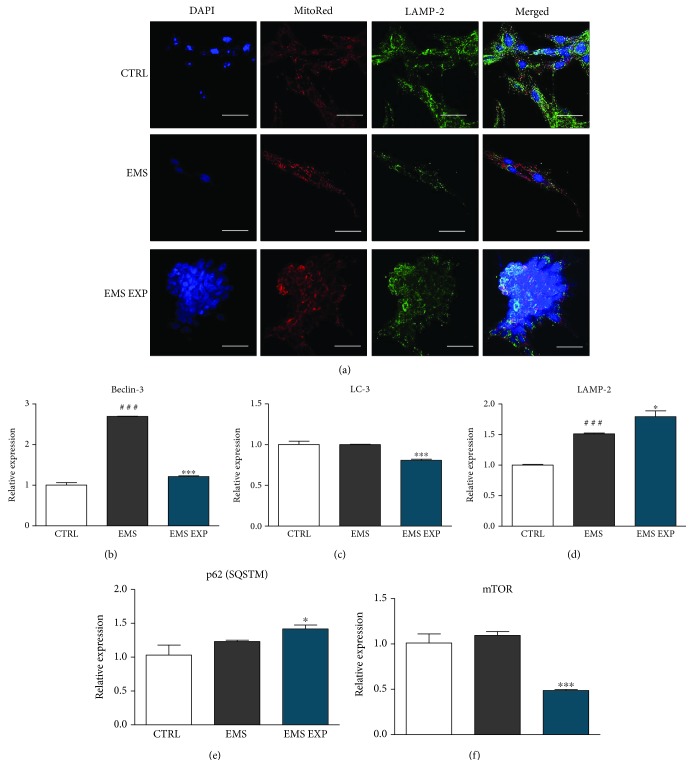
Investigation of autophagy during early chondrogenesis. Prior to experiments, cells were pretreated with AZA/RES for 24 hours, then the experimental medium was replaced by osteogenic differentiation medium. Cells were cultured in that medium for five days and after were subjected to further analysis. Nuclei of cells were stained with DAPI. Mitochondria were stained with MitoRed dye while LAMP-2 was visualized using immunofluorescence. Mitochondrial net and LAMP-2 were visualized in cells using confocal microscopy (a). Expression of genes related to autophagy was investigated with RT-PCR. Expression of Beclin-3 (b) and LC3 (c) was diminished in EMS EXP; however, LAMP-2 (d) and p62 (e) mRNA levels were upregulated in that group. Similarly, mTOR expression was diminished (f). Results expressed as mean ± S.D. Statistical significance indicated as an asterisk (^∗^) when comparing the result to ASC_EMS_ and as a number sign (#) when comparing to ASC_CTRL_. ^∗^*p* < 0.05; ^###^ and ^∗∗∗^*p* < 0.001.

To assess autophagy during the early stage of chondrogenic differentiation, confocal microscopy analysis was performed (Figure 6(a)). Cells were stained using the MitoRed dye and anti-LAMP-2 antibodies. Images taken from the control and EMS group presented robust mitochondrial network located evenly within the cytoplasm. Mitochondria in AZA/RES-treated ASC_EMS_ were located in the perinuclear area. Interestingly, the immunofluorescence staining for LAMP-2 revealed increased lysosome formation in both the control and AZA/RES group. In the experimental group, mitochondria and lysosomes were located around the nucleus. Merged pictures showed that the control group contains much more lysosomes in comparison to the other groups. Interestingly, lysosomes containing mitochondria occurred the most frequently in the control group. In addition, autophagy was investigated using the RT-PCR technique. We observed a significant decrease mRNA level of Beclin-3 (Figure 6(b)), LC3 (Figure 6(c)), p62 (Figure 6(e)), and mTOR (Figure 6(f)) (*p* < 0.001) in ASC_EMS_ after treatment with AZA/RES.

### 3.7. Evaluation of Mitochondrial Net Types

**Figure 7:**
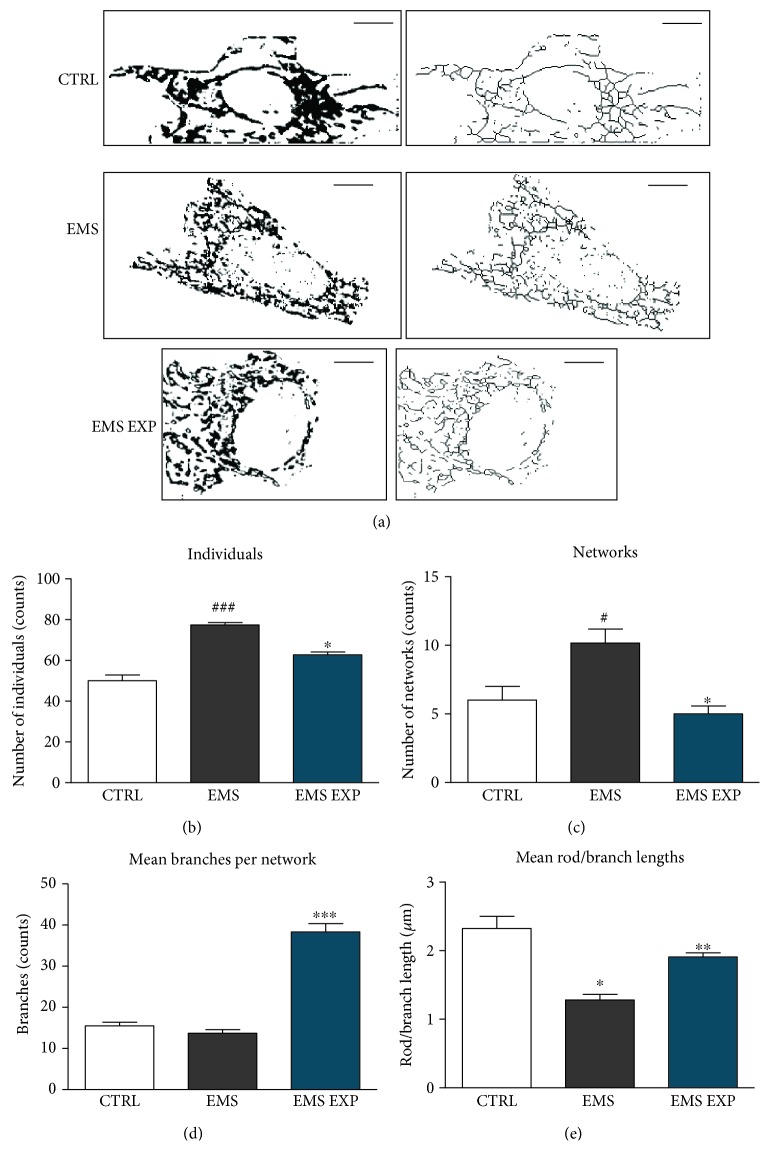
Evaluation of mitochondrial net types. Prior to experiments, cells were pretreated with AZA/RES for 24 hours, then the experimental medium was replaced by osteogenic differentiation medium. Cells were cultured in that medium for five days and after were subjected to further analysis. Mitochondrial net visualization was performed in the Mitochondrial Network Analysis (MiNA) toolset using the photographs from MitoRed staining (a). Obtained images were further quantified in the same software. The mean number of both individuals (b) and networks (c) was substantially greater in EMS than in EMS_EXP_. The number of mean branches per network was significantly increased in EMX_EXP_ (d). The ratio of rod to branch was diminished in EMS cells; however, after treatment with AZA/RES, increased value of that parameter was noted (e). Results expressed as mean ± S.D. Statistical significance indicated as an asterisk (^∗^) when comparing the result to ASC_EMS_ and as a number sign (#) when comparing to ASC_CTRL_. ^#^ and ^∗^*p* < 0.05; ^∗∗^*p* < 0.01; ^###^ and ^∗∗∗^*p* < 0.001.

Formation and maintenance of a mitochondrial network play a major role in maintaining mitochondrial function. Mitochondrial net analysis was performed in the MiNA software, and representative images are shown in Figure 7(a). AZA/RES treatment decreased the number of small, single mitochondria in comparison to ASC_EMS_ (Figure 7(b)). In addition, treated cells exhibited strong decrease in the number of mitochondrial networks in comparison to the EMS group (Figure 7(c), *p* < 0.05). However, mean amount of branches (Figure 7(d)) and mean rod to branch length ratio (Figure 7(e)) were significantly increased after AZA/RES treatment in comparison to untreated cells.

### 3.8. Evaluation of Mitochondrial Dynamics during Chondrogenic Differentiation

**Figure 8:**
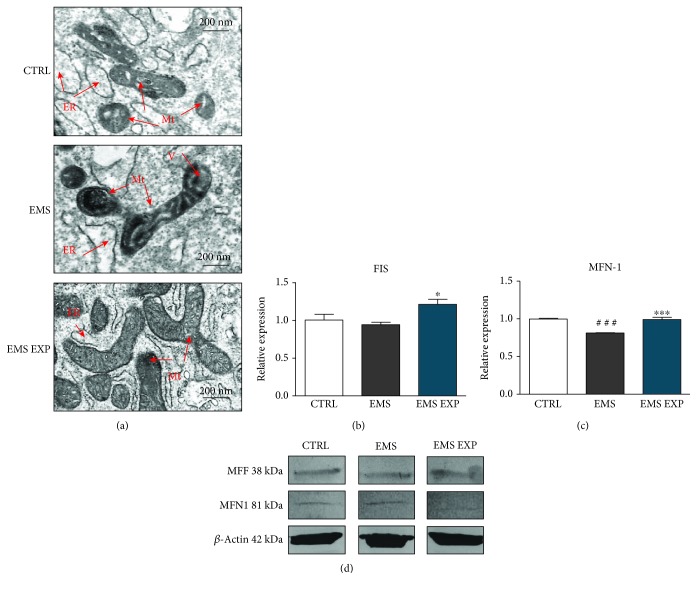
Evaluation of mitochondrial dynamics during chondrogenic differentiation. Prior to experiments, cells were pretreated with AZA/RES for 24 hours, then the experimental medium was replaced by osteogenic differentiation medium. Cells were cultured in that medium for five days and after were subjected to further analysis. Mitochondrial morphology was visualized using TEM (a). Mitochondria from ASC_EMS_ were characterized by disarrayed cristae and vacuole formation. Furthermore, expression of FIS (b) and MFN (c) was evaluated using RT-PCR. Amount of MFF and MFN protein cell lysates was established with western blot (d). Abbreviations: Mt: mitochondria, ER: endoplasmic reticulum, V: vacuole. Results expressed as mean ± S.D. Statistical significance indicated as an asterisk (^∗^) when comparing the result to ASC_EMS_ and as a number sign (#) when comparing to ASC_CTRL_. ^∗^*p* < 0.05; ^###^ and ^∗∗∗^*p* < 0.001.

Mitochondrial dynamics was determined using the TEM microscopy. Mitochondria in ASC_CTRL_ during chondrogenic differentiation contained several vacuoles. Moreover, the cristae were shorter or missing. Mitochondria in ASC_EMS_ exhibited several alternations including vesiculation and missing of cristae, as well as a shape deterioration (Figure 8(a)). Mitochondria in ASC_EMS EXP_ presented elongated or round morphology with thick cristae. In addition, quantitative analysis of FIS (Figure 8(b)) and MFN (Figure 8(c)) mRNA levels using RT-PCR revealed a significant overexpression of those genes in ASC_EMS_ after treatment with AZA/RES (*p* < 0.05 and *p* < 0.001, respectively) in comparison to the control and EMS groups. Overexpression of MFN and branching shape suggest mitochondrial fusion while high amount of FIS mRNA and presence of small round mitochondria are indicative of mitochondrial fragmentation. Moreover, western blotting analysis showed no differences of MFF protein expression between experimental groups, while the level of MFN1 was lower in AZA/RES treated ASC_EMS_ compared to both ASC_CTRL_ and ASC_EMS_ (Figure 8(d)).

### 3.9. Evaluation of Mitophagy during Chondrogenic Differentiation

**Figure 9:**
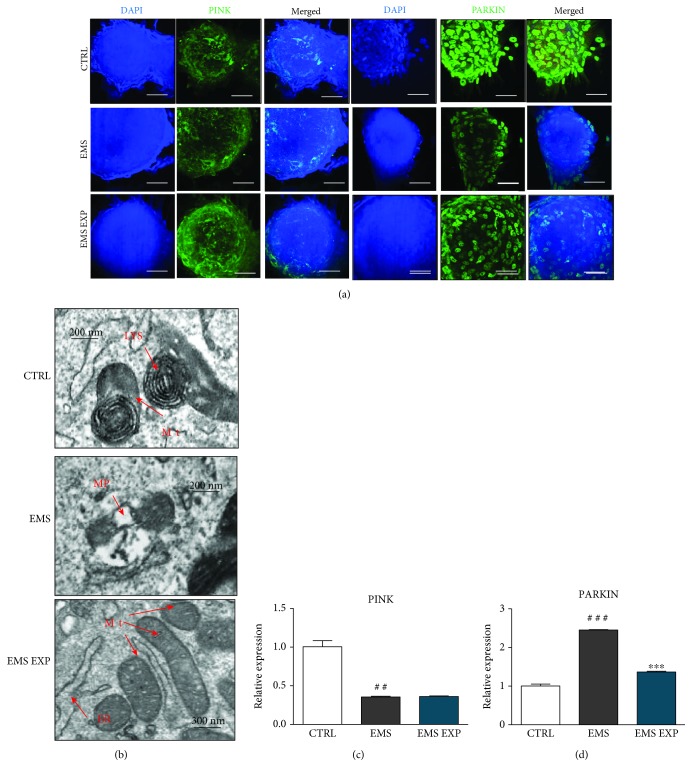
Evaluation of mitophagy during chondrogenic differentiation. Prior to experiments, cells were pretreated with AZA/RES for 24 hours, then the experimental medium was replaced by osteogenic differentiation medium. Cells were cultured in that medium for five days and after were subjected to further analysis. Using immunofluorescence, localization of PINK and PARKIN in chondrogenic nodules was visualized with confocal microscope (a). Nuclei of cells were stained with DAPI (a). PINK and PARKIN fluorescence was diminished in EMS cells as well as size of nodules. Furthermore, formation of mitophagosomes and lysosomes was established with TEM (b). Mitochondria in the EMS cells were packed into mitophagosomes while mitochondria from the EMS EXP groups did not display morphology abnormalities. Interestingly, mitochondria in the CTRL group frequently fused with lysosomes. Expression of PINK (c) and PARKIN (d) was investigated with RT-PCR. Abbreviations: LYS: lysosome, Mt: mitochondrion, MP: mitophagosome, ER: endoplasmic reticulum. Results expressed as mean ± S.D. Statistical significance indicated as an asterisk (^∗^) when comparing the result to ASC_EMS_ and as a number sign (#) when comparing to ASC_CTRL_. ^##^*p* < 0.01, ^###^ and ^∗∗∗^*p* < 0.001.

Mitophagy, crucial process for the removal of deteriorated organelles, was investigated by immunofluorescence for PINK and PARKIN (Figure 9(a)). Obtained images indicated on increased PINK levels in ASC pretreated with AZA/RES. Fluorescent intensity originating from PARKIN was diminished in ASC_EMS_; however, in cells from experimental group, greater signal was noted. TEM photographs showed mitochondrial-lysosomal fusion in the healthy cells, what suggests mitophagy occurs during chondrogenesis (Figure 9(b)). Similarly, cells isolated from EMS individuals displayed mitophagosome formation. However, AZA/RES treatment attenuates mitophagy occurs in the EMS group. In addition, we observed decreased expression of PINK gene in cells pretreated with AZA/RES (Figure 9(c)). Moreover, analysis of PARKIN expression revealed increased mRNA level of this gene in ASC_EMS_ while decreased in the experimental group (Figure 9(d)).

### 3.10. Epigenetic Alternations during Chondrogenesis in Control and Experimental Condition

**Figure 10:**
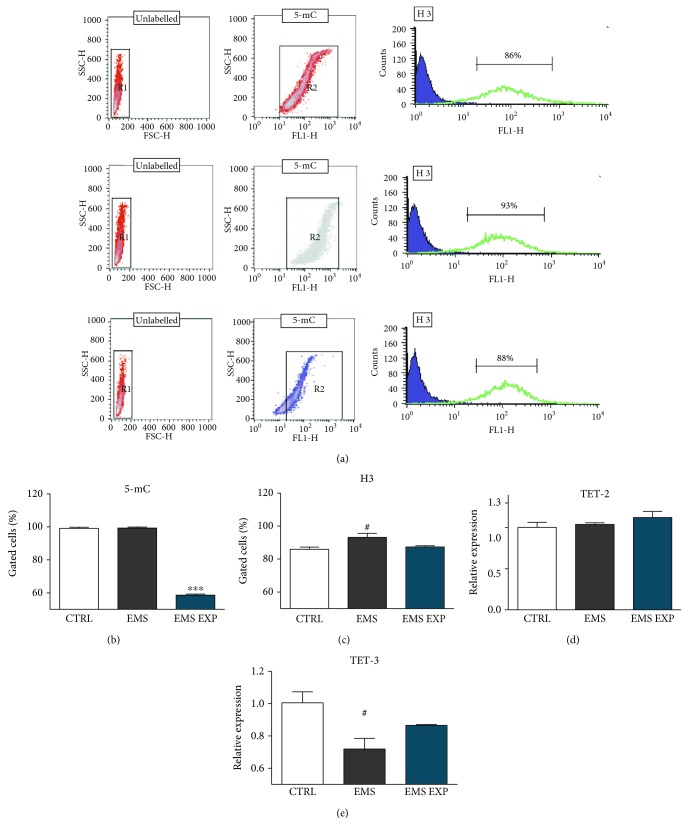
Epigenetic alternations during chondrogenesis in control and experimental conditions. Prior to experiments, cells were pretreated with AZA/RES for 24 hours, then the experimental medium was replaced by osteogenic differentiation medium. Cells were cultured in that medium for five days and after were subjected to further analysis. Epigenetic alternations in cells were investigated with flow cytometry. Representative graphs show results of 5-mC and H3 accumulation in cells (a). Obtained results revealed decreased levels of 5-mC in EMS EXP (b). Increased H3 amount was noted in the EMS group (c). Expression of TET-2 (d) and TET-3 (e) was analyzed with RT-PCR. Results expressed as mean ± S.D. Statistical significance indicated as an asterisk (^∗^) when comparing the result to ASC_EMS_ and as a number sign (#) when comparing to ASC_CTRL_. ^#^*p* < 0.05, ^∗∗∗^*p* < 0.001.

In order to assess the effects of AZA/RES treatment on ASC_EMS_, flow cytometry analysis was performed (Figure 10(a)). Our results demonstrated a gradual decrease of 5-mC in the treated cells in comparison to both control and EMS groups (Figure 10(b), *p* < 0.001). Expression of H3 was elevated in EMS cells (Figure 10(c)). Additionally, RT-PCR analysis demonstrated overexpression of both TET-2 (Figure 10(d)) and TET-3 (Figure 10(e)) in ACS_EMS_ treated with AZA/RES; however, the differences had no statistical significance.

### 3.11. Evaluation of Anti-Inflammatory Properties of Prechondroblastic Cells

**Figure 11:**
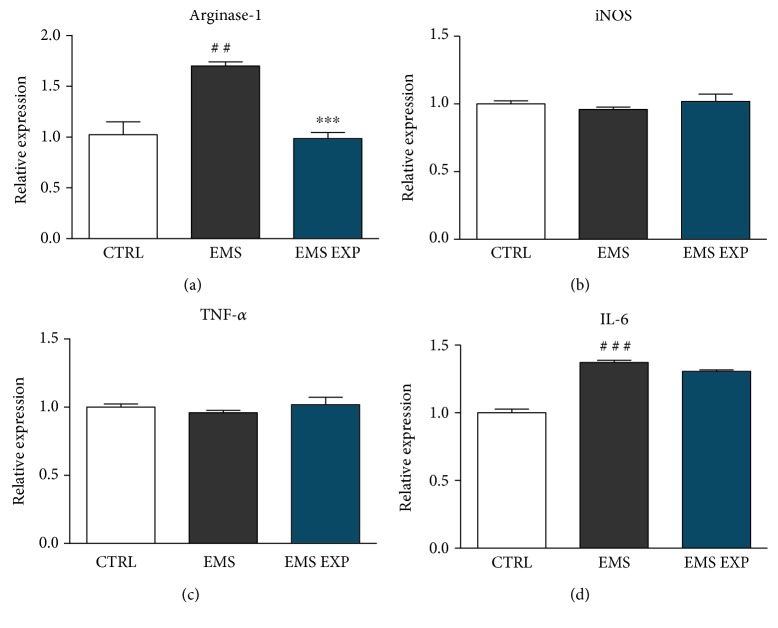
Evaluation of anti-inflammatory properties of prechondroblastic cells. The effect of AZA/RES on the activation status of RAW 264.7 macrophages. RAW 264.7 were seeded at a density of 1 × 106 cells/mL, and after 18 hours, not-adherent cells were removed. Next, LPS was added to the culture media at a concentration of 1 *μ*g/mL, and the experiment was continued for another 24 h. At the same time, ASCs after the fifth day of differentiation in the amount of 4 × 10^4^ were added to culture wells. After 24 hours of coculture, media were collected for analysis of the macrophages' secretory activity, and the cells were lysed by adding TRI Reagent. Arginase-1 (a), iNOS (b), TNF-*α* (c), and IL-6 (d) gene expression was evaluated in macrophages with RT-PCR. Results expressed as mean ± S.D. Statistical significance indicated as an asterisk (^∗^) when comparing the result to ASC_EMS_ and as a number sign (#) when comparing to ASC_CTRL_. ^##^*p* < 0.05, ^###^ and ^∗∗∗^*p* < 0.001.

Results of RT-PCR analysis showed decreased amounts of Arginase-1 in macrophages cocultured with ASC_EMS_ treated with AZA/RES (Figure 11(a)). There were no significant differences in the expression of iNOS (Figure 11(b)) and TNF-*α* (Figure 11(c)) between investigated groups. IL-6 was significantly elevated in the EMS group in comparison to control; however, AZA/RES treatment did not influence IL-6 expression.

## 4. Discussion

In the presented study, we revealed that the combination of AZA and RES (AZA/RES) enhance the chondrogenic differentiation potential of ASC_EMS_ through modulation of autophagy, mitochondrial dynamics, and reduction of ER stress. In our previous work, we have shown that EMS impairs multilineage differentiation potential of ASC through excessive accumulation of oxidative stress factors, which is associated with the deterioration of mitochondrial metabolism and dynamics [16–18, 41]. In consequence, clinical application of ASC_EMS_ in the treatment of EMS is limited. Thus, we hypothesized that proper pharmacotherapy of those cells in vitro based on the application of AZA/RES may reverse aged phenotype and improve multipotency of those cells. In consequence, rejuvenated cells may become an effective therapeutic tool in EMS treatment.

During metabolic syndrome, proinflammatory microenvironment of adipose tissue negatively affects ASC population residing within it [3, 33]. Here, we showed that *ex vivo* application of AZA/RES in ASC_EMS_ enhanced the expression of prochondrogenic genes including vimentin, decorin, and SOX-9. Enhanced expression of SOX-9 stimulates the formation of extracellular matrix and helps chondrocytes maintain their phenotype, protecting them against hypertrophy. Obtained data stands with a good agreement with Lei and colleagues, who revealed a positive effect of RES on chondrogenic differentiation potential in bone marrow-derived mesenchymal stem cells (BMSC) through the activation of the SIRT1 pathway [42]. Here, we observed increased mRNA expression of COMP accompanied by the increased proliferation rate, number, and size of chondrogenic nodules in cells treated with AZA/RES. Using RT-PCR, we showed that ASC_EMS_ treated with AZA/RES displayed diminished expression of the proapoptotic p21 transcript when compared to nontreated cells and that the expression of the antiapoptotic gene BCL-2 correlated with enhanced expression of Tet methylcytosine dioxygenase 2 and 3 (TET2 and TET3). It has been recently shown that TET proteins are catalytically capable of oxidizing DNA 5-methylcytosine (5-mC) to 5-hydroxymethylcytosine (5-hmC) which is a necessary step in the complete removal of the methylated cytosine [43]. Those findings stand with a good agreement with our data as we observed decreased amount of 5-mC in AZA/RES-treated ASC_EMS_ when compared to nontreated cells. Epigenetic changes in both embryonic as well as mesenchymal stem cells were shown to be crucial for osteogenic and chondrogenic differentiation [44, 45]. Our previous data demonstrated that application of AZA in aged mesenchymal stem cells reverse epigenetic alternations, apoptosis, and oxidative stress in those cells [29]. Chondroprotective, antiapoptotic, antisenescent, and antiaging effect of AZA and RES was documented *in vitro* and *in vivo* in multiple other studies [46–48].

Excessive accumulation of oxidative stress factors in ASC was shown to diminish their multilineage differentiation potential and ability to form colonies, induce senescence and aging, disturb mitochondrial metabolism and dynamics, trigger ER stress, and cause autophagy impairment [49, 50]. Here, we observed that ASC_EMS_ treated with AZA/RES displayed increased MMP, enhanced SOD activity, reduced ER stress, and upregulated p62 and LAMP-2 expression. Interestingly, we noted in those cells increased NO levels, which contributed to the improvement of chondrogenic differentiation and enhanced expression of SOX-9 and COPM [51]. Recent research of Kang and colleagues [52] demonstrated that the deficiency of cartilage-specific autophagy played a critical role in ER stress and chondrogenesis in a PERK-ATF4-CHOP-dependent manner. It is important in the context of our results, as we demonstrated that AZA/RES induced autophagy in ASC_EMS_ and improved the formation of cartilage extracellular matrix. Qin and colleagues reported that intra-articular injection of RES delayed cartilage degeneration in C57BL/6 mice by inducing autophagy via silencing the mTOR pathway [53]. In our previous study, we showed that autophagy deterioration can be considered as one of the possible mechanisms that trigger apoptotic/senescence phenotype in ASC_EMS_, thereby impairing their differentiation potential. Here, we found that ex vivo application of AZA/RES enhanced autophagy in ASC_EMS_ during early chondrogenesis resulting in improvement of their differentiation potential.

Considering that AZA/RES protects ASC against aged phenotype, improves antioxidant defence against free radicals, and enhances chondrogenic differentiation by modulating autophagy, it is tempting to speculate that it also affects mitochondrial biogenesis and dynamics. Here, the observed enhanced expression of FIS and MFN-1 in AZA/RES-treated ASC_EMS_ which indicated on profound effects of mitochondrial dynamics on the differentiation process. Our observations are in line with recent findings published by Forni and colleagues [54], who showed that mitochondrial fragmentation associated with higher expression of FIS and enhanced autophagy was accompanied by profound bioenergetic alterations during the commitment period. TEM and confocal microscopy combined with western blot analysis enabled us to visualize mitochondria and establish their dynamics in the early stage of chondrogenesis. In our previous study [16], we observed that the EMS negatively affects the ultrastructure of mitochondria in ASC. Here, we revealed that the application of AZA/RES improved the ultrastructure of these organelles. As mitochondria play a central role in regulating cell fate, their proper functionality is crucial for effective differentiation. Upon differentiation, mitochondria become activated by yet unknown mechanism, leading to prevalence of oxidative phosphorylation after glycolysis. Furthermore, we analyzed mitochondrial network and revealed increased number of branches per network, which indicates on the occurrence of mitochondrial fission. Moreover, we found a higher number of mitochondria in the experimental group, which may correspond to increased biogenesis in AZA/RES-treated cells. ROS accumulation is another essential factor in the differentiation process. It was shown that ROS levels were increased during chondrocyte development [55–58]. Although extensive research has been carried out on the chondrogenic differentiation of MSCs, the role of mitochondrial dynamics and ROS still needs further clarification. Modulation of mitochondrial dynamics may lead to the improvement of differentiation process, because it plays a central role in directing stem cell fate. Furthermore, Kim et al. [59] proved that ROS accumulation is essential for survival and differentiation in the early events of chondrogenic differentiation. It also correlated with our results, as we observed increased ROS levels in the AZA/RES-treated group, which resulted in enhanced chondrogenic differentiation.

In summary, proliferation rate, viability, and antioxidant defence were improved by in vitro application of AZA/RES, which underlines the beneficial effect of that chemicals in the course of chondrogenic differentiation. Moreover, the application of AZA/RES induced autophagy and improved mitochondrial dynamics during chondrogenic differentiation, which was accompanied by improved mitochondrial functionality. Further researches are necessary to identify and explore mechanisms that are involved in ASC_EMS_ quiescence and lead to the impairment of multilineage differentiation; this is important for the successful development of autologous cell therapy of EMS.

## Figures and Tables

**Table 1:** Criteria for horse classification. This table is reproduced from Kornicka et al., 2017 (under the Creative Commons Attribution License/ https://www.ncbi.nlm.nih.gov/pmc/articles/PMC6307768/).

Group	Age	Baseline serum insulin (mIU/mL)	Insulin (mIU/mL) 60 min postoral sugar administration	Baseline glucose (mg/dL)	CGIT:GLU in 45 min (mg/dL)	BCS	CNS	LEP (ng/mL)	BW (kg)
EMS	12 ± 2	180 ± 20	260 ± 11	72 ± 11	163 ± 26	7.2 ± 0.2	3.9 ± 0.7	4.8 ± 1.2	680 ± 30
Control	11 ± 2	13 ± 4	36 ± 3	84 ± 7	88 ± 5	6.4 ± 0.3	1.9 ± 0.6	1.1 ± 0.6	590 ± 20

Bw: body weight; BCS: body condition score; CNS: cresty neck score; CGIT: combined glucose-insulin test; LEP: leptin; GLU: glucose.

**Table 2:** Sequences of primers used for RT-PCR.

Gene name	Forward and reverse primer sequence (5′-3′)	Size of amplicon (bp)	Accession no.
Vimentin	F: GCAGGATTTCTCTGCCTCTT	203	XM_014846038.1
	R: TATTGCTGCACCAAGTGTGT		
Decorin	F: GATGCAGCTAGCCTGAGAGG	248	XM 014841263.1
	R: GTGTTGTATCCAGGTGGGCA		
COMP	F: AGTGTCGCAAGGATAACTGCGTGA	238	NM_001081856.1
	R: TCCTGATCTGTGTCCTTCTGGTCA		
SOX-9	F: GAACGCCTTCATGGTGTGGG	225	XM_014736619.1
	R: TTCTTCACCGACTTCCTCCG		
p53	F: TACTCCCCTGCCCTCAACAA	252	U37120.1
	R: AGGAATCAGGGCCTTGAGGA		
p21	F: GAAGAGAAACCCCCAGCTCC	241	XM_014853747.1
	R: TGACTGCATCAAACCCCACA		
Caspase-3	F: GGCAGACTTCCTGTATGCGT	167	XM_023630401.1
	R: CCATGGCTACCTTGCGGTTA		
Bcl-2	F: TTCTTTGAGTTCGGTGGGGT	164	XM_014843802.1
	R: GGGCCGTACAGTTCCACAA		
BAX	F: TTCCGACGGCAACTTCAACT	150	XM 005607505.1
	R: GGTGACCCAAAGTCGGAGAG		
CHOP	F: AGCCAAAATCAGAGCCGGAA	272	XM 014844003.1
	R: GGGGTCAAGAGTGGTGAAGG		
PERK	F: GTGACTGCAATGGACCAGGA	283	XM 014852775.1
	R: TCACGTGCTCACGAGGATATT		
eIF2*α*	F: AGTCTTCAGGCATTGGCTCC	489	XM_001488848.6
	R: CCGAGTGGGACATGTATCGG		
Beclin-3	F: GATGCGTTATGCCCAGATGC	233	XM 014833759.1
	R: AACGGCAGCTCCTCTGAAAT		
LAMP-2	F: GCACCCCTGGGAAGTTCTTA	139	XM 014733098.1
	R: ATCCAGCGAACACTCTTGGG		
p62 (SQSTM)	F: CATCGGAGGATCCCAGTGTG	207	XM_005599173.3
	R: CCGGTTTGTTAGGGTCGGAA		
LC3	F: TTACTGCTTTGCTCTGCCAC	213	XM 005608485.2
	R: AGCTGCTTCTCCCCCTTGT		
mTOR	F: GGGCAGCATTAGAGACGGTG	221	XM 005607537.2
	R: ATGGTTGATTCGGTGTCGCA		
TET-2	F: ATCCTGATCCTGGTGTGGGA	143	XM_023636796.1
	R: CCTTGACAGGCACAGGTTCT		
TET-3	F: CAGCCTGCATGGACTTCTGT	188	XM_023618871.1
	R: GTTCTCCTCACTGCCGAACT		
FIS	F: GGTGCGAAGCAAGTACAACG	118	XM 001504462.4
	R: GTTGCCCACAGCCAGATAGA		
PINK	F: GCACAATGAGCCAGGAGCTA	298	XM 014737247.1
	R: GGGGTATTCACGCGAAGGTA-0		
PARKIN	F: TCCCAGTGGAGGTCGATTCT	218	XM 014858374.1
	R: CCCTCCAGGTGTGTTCGTTT		
MFN1	F: AAGTGGCATTTTTCGGCAGG	217	XM 001495170.5
	R: TCCATATGAAGGGCATGGGC		
Arginase-1	F: CCAGAAGAATGGAAGAGTCAGTGT	252	NM_007482.3
	R: GCAGATATGCAGGGAGTCACC		
iNOS	F: GACAAGCTGCATGTGACATC	325	XM_006532446.3
	R: GCTGGTAGGTTCCTGTTGTT		
TNF-*α*	F: ACAGAAAGCATGATCCGCGA	295	NM_013693.3
	R: CTTGGTGGTTTGCTACGACG		
IL-6	F: GAGGATACCACTCCCAACAGACC	146	NM_001314054.1
	R: AAGTGCATCATCGTTGTTCATACA		
GAPDH	F: GATGCCCCAATGTTTGTGA	250	NM 001163856.1
	R: AAGCAGGGATGATGTTCTGG		
Beta actin	CATACGCCTGCAGAGTTAAGCA	73	NM_009735.3
	GATCACATGTCTCGATCCCAGTAG		

Sequences: amplicon length and access numbers of the primer sets. COMP: cartilage oligomeric matrix protein; Sox-9: transcription factor SOX-9; p21: cyclin-dependent kinase inhibitor 1A; p53: tumor suppressor p53; BCL-2: B-cell lymphoma 2; BAX: BCL-2-associated X protein; CHOP: DNA damage inducible transcript 3; PERK: PRKR-like endoplasmic reticulum kinase; eIF*α*: eukaryotic initiation factor 2; Beclin-3: autophagy related (BECN1); LAMP2: lysosomal-associated membrane protein 2; p62: Sequestosome-1; LC3: microtubule associated protein 1 light chain 3 beta (MAP1LC3B); mTOR: mammalian target of rapamycin; TET 2: Tet methylcytosine dioxygenase 2; TET 3: Tet methylcytosine dioxygenase PINK: PTEN-induced putative kinase 1 (PINK1); PARKIN: parkin RBR E3 ubiquitin protein ligase (PARK2); FIS: mitochondrial fission 1 molecule; MFN1: mitofusin 1; iNOS: inducible nitric oxide synthase; TNF-*α*: tumor necrosis factor alpha; IL-6: interleukin 6; GADPH: glyceraldehyde-3-phosphate dehydrogenase.

## Data Availability

The data used to support the findings of this study are available from the corresponding author upon request.
